# Machine Learning for Long Cycle Maintenance Prediction of Wind Turbine

**DOI:** 10.3390/s19071671

**Published:** 2019-04-08

**Authors:** Chia-Hung Yeh, Min-Hui Lin, Chien-Hung Lin, Cheng-En Yu, Mei-Juan Chen

**Affiliations:** 1 Department of Electrical Engineering, National Sun Yat-sen University, Kaohsiung 80424, Taiwan; d063010005@student.nsysu.edu.tw (M.-H.L.); alleneatpie@gmail.com (C.-E.Y.); 2 Department of Electrical Engineering, National Taiwan Normal University, Taipei 10610, Taiwan; 3 Taiwan Power Company, Kaohsiung 80446, Taiwan; u621363@taipower.com.tw; 4 Department of Electrical Engineering, National Dong Hwa University, Hualien 97401, Taiwan; cmj@gms.ndhu.edu.tw

**Keywords:** Internet of Things (IoT), sensors, deep learning, data mining, long cycle maintenance, convolutional neural network, wind turbine, conditional monitoring

## Abstract

Within Internet of Things (IoT) sensors, the challenge is how to dig out the potentially valuable information from the collected data to support decision making. This paper proposes a method based on machine learning to predict long cycle maintenance time of wind turbines for efficient management in the power company. Long cycle maintenance time prediction makes the power company operate wind turbines as cost-effectively as possible to maximize the profit. Sensor data including operation data, maintenance time data, and event codes are collected from 31 wind turbines in two wind farms. Data aggregation is performed to filter out some errors and get significant information from the data. Then, the hybrid network is built to train the predictive model based on the convolutional neural network (CNN) and support vector machine (SVM). The experimental results show that the prediction of the proposed method reaches high accuracy, which helps drive up the efficiency of wind turbine maintenance.

## 1. Introduction

Internet of Things (IoT) sensors can communicate and interact over the Internet, and be monitored and controlled remotely [1,2]. Data collected from sensors are to be processed to generate valuable information for management. Predictive maintenance techniques are designed to help evaluate the equipment condition to determine the maintenance schedule [3,4]. We aim to predict when the wind turbines would fail and how long they would last before their failure.

Wind power, different from burning fossil fuels, is plentiful, clean, and renewable. People have made use of wind power for hundreds of years. Wind turbines are usually operated in tough environments and need to endure high wind speed and extreme temperatures. The maintenance cost of wind turbine components is usually higher than the procurement cost. A wind farm would encounter many difficulties such as drivetrain failures, spalled bearings, and fractured gears, so its operation and maintenance have become an important research field.

The research fields regarding maintenance cost reduction include periodic inspections, corrective maintenance, and condition monitoring. [5]. Condition monitoring is normally used on rotating machinery, so it is very suitable for monitoring wind turbines [6]. Since condition monitoring is often used to maintain the wind turbines in the wind power industry, data collected from sensors are analyzed to predict the equipment condition. When an unusual event is detected, sensors record the event using event codes, which helps the operator to quickly understand the wind turbines’ status at that time. Because there are erroneous and irrelevant data in the large amount of data collected, efficient data pre-processing and analyses techniques are used to extract valuable information for maintenance. However, most of the methods are rule-based, and it is difficult for these methods to reach high accuracy of maintenance cycle prediction. This paper tries to analyze the collected data to improve the efficiency of wind turbine maintenance. Among all maintenance categories, the most time-consuming task is the long cycle maintenance, which requires a great deal of human and material resources. Therefore, the efficient and effective prediction of the occurrence of long cycle maintenance is very important in the management of wind turbines.

In Changhua Coastal Industrial Park, there are two wind farms where all 31 wind turbines are installed with the V80 module [7]. The sensors include an anemometer, electricity meter, gearbox thermometer, and high-speed bearings. A large amount of data is collected by the sensors when monitoring the wind turbines, and data mining techniques [8] are then employed to process the massive amount of data and dig out useful information for maintenance. In our method, all maintenance periods are firstly divided into four categories and the relations of the maintenance periods of these categories are analyzed. The Apriori algorithm [9] is used to find the key event codes, which occur before the maintenance periods. Also, linear regression is applied to filter some errors from the operation data. Six features: wind speed, the power output of wind turbines, the oil temperature of the wind turbine gearbox, the temperature of the high-speed bearings, the number of consecutive short under-maintenance periods, and the frequency of the event codes, are extracted from the results above. Then a hybrid network consisting of a convolutional neural network (CNN) and support vector machine (SVM) is constructed to predict the long cycle maintenance time. Experimental results show that the proposed method achieves high accuracy in predicting the period of long cycle maintenance.

The rest of this paper is organized as follows. In Section 2, we review the background knowledge. Section 3 focuses on the proposed method of long cycle maintenance. In Section 4, the experimental results demonstrate the efficiency of the proposed method. Concluding remarks and future work are given in Section 5.

## 2. Background Review

### 2.1. Condition Monitoring of Wind Turbines

Condition monitoring is the process of monitoring the condition of machinery to identify a significant change which is implicative of an operational problem in order to prevent its failure and the consequences associated with it. Condition monitoring uses sensors and signal-processing equipment to continuously monitor wind turbine components regarding vibration analysis [10], oil analysis [11], performance monitoring [12], strain measurement [13], acoustic emission [14], and ultrasonic testing techniques [15]. Through data mining techniques, the data collected during the operation of major components can be used to reduce major component failure. Maintenance schedules can be planned more efficiently, and the costs of maintenance are reduced. Condition monitoring is commonly used in most offshore wind farms because of their high costs of maintenance.

### 2.2. Convolutional Neural Network (CNN)

Deep learning is a technique required for implementing machine learning. CNNs are one of the most popular architectures in deep learning. CNNs are hierarchical neural networks and have been successfully used for data analyses, speech recognition, image classification, pattern recognition, and computer vision. A typical CNN structure contains subsampling layers between each convolutional layer and fully connected layers. In order to implement the CNN, there are several parameters to be set, such as input size, kernel (filter) dimensions, number of neurons in each layer, and the topology of the CNN. In this paper, CNNs are trained to learn the features that are related to wind turbine maintenance. A combination of the CNN and SVM (Support Vector Machine), where the output of the CNN is replaced by an SVM, could achieve great performance for classification.

### 2.3. Simple Linear Regression

In statistics, simple linear regression is a method which allows us to summarize and study the relationships between two continuous variables. Consider the model function for the slope β and y-intercept α in the simple linear regression:

(1) $$y=\alpha +\beta x+\mu_{i},$$

where $\mu_{i}$, named residuals, are the distance between the points of the data and the regression line. The advantage of simple linear regression is that it is efficient and intuitive to find the nature of the relationship between two variables. In this paper, the simple linear regression is used to model the relationship between the operation data.

### 2.4. Apriori Algorithm

Data mining is the computational process of finding patterns in large data sets. Some techniques have been proposed in previous research regarding data mining on wind turbines, including prediction models for wind turbine states [16], fault diagnosis [17,18], and power curve monitoring [19]. The Apriori algorithm is one of the most popular algorithms in discovering association rules. The Apriori algorithm was proposed by Srikant et al. in 1994 and is designed to work on transactional databases. The goal of this algorithm is to extract information from a data set and transform it into a comprehensible structure for further use. The simple description of the Apriori algorithm is as follows. Assume that there are two itemsets X and Y in the database. If there is an association rule between X and Y, the rule is denoted as X⇒Y called association rules.

Support: a representation of how frequently the itemset appears in the database, “Support” is defined as:

(2) Support{X⇒Y}=P(X∩Y).

Confidence: the transaction that contains X would also contain Y, “Confidence” is defined as:

(3) $$Confidence\left\{X\Rightarrow Y\right\}=P\left(Y|X\right)=\frac{P\left(X\cap Y\right)}{P\left(X\right)}.$$

Lift: the ratio of the confidence of the association rule to the probability of Y, “Lift” is defined as:

(4) $$Lift\left\{X\Rightarrow Y\right\}=\frac{P(Y|X)}{P\left(Y\right)}=\frac{P\left(X\cap Y\right)}{P\left(X\right)P\left(Y\right)}.$$

If Lift{X⇒Y}>1, X and Y are dependent, which makes the rule potentially useful in predicting. The Apriori algorithm generates candidate item sets of the length k from item sets of the length k−1. Then it reduces the candidates which have infrequent sub-patterns. After that, it scans the database to determine frequent item sets among the candidates. Apriori utilizes a “bottom-up” approach to extend frequent subsets of one item at a time (a step known as candidate generation), and groups of candidates are tested against the data. The algorithm ends when no further successful extensions are found. In this paper, we employ the Apriori algorithm to find the key event codes of wind turbines.

## 3. Proposed Method

In this section, maintenance time data, event codes, and operation data are introduced first, followed by the explanations on the proposed methods: Data aggregation and prediction model construction.

### 3.1. Data Aggregation

**Figure 1:**
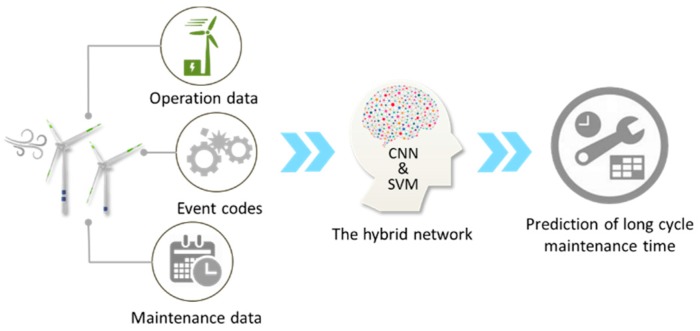
The architecture of the long cycle maintenance time prediction.

Figure 1 illustrates the concept of the proposed method and Table 1 shows the specifications of the V80 module wind turbines in Changhua Coastal Industrial Park. Firstly, all the data are aggregated to filter errors and extract significant features. Data aggregation is a process in which information is gathered and expressed in a summary form for the purpose of the follow-up statistical analysis. The results of data aggregation will be applied to deep learning models to predict the long cycle maintenance time.

#### 3.1.1. Maintenance Time and Event Codes

**Figure 2:**
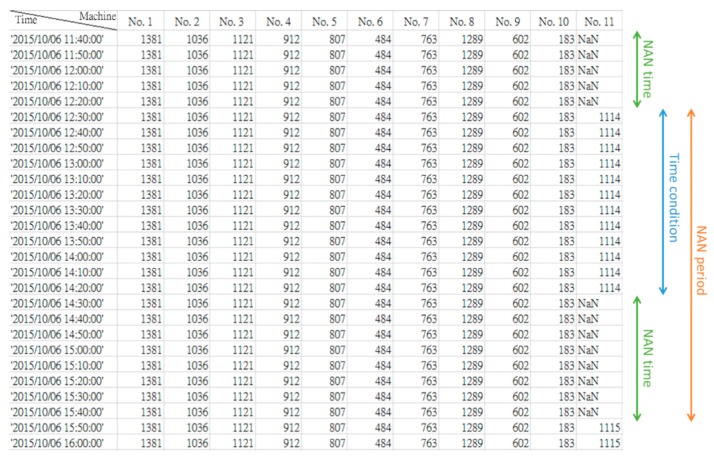
Recorded maintenance time data.

**Figure 3:**
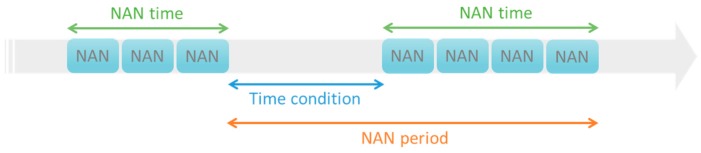
An illustration of the relationships between NAN time, time condition, and NAN period.

**Figure 4:**
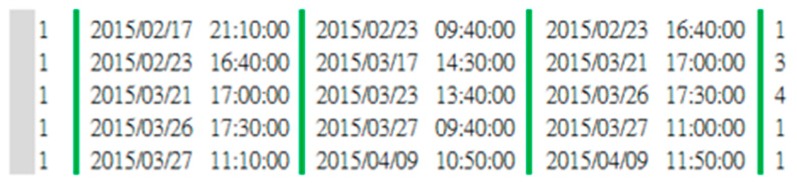
Example of data after the data aggregation step.

Maintenance time data archives the records of accumulated maintenance hours and maintenance status. The maintenance time data are collected for 941 consecutive days, and the frequency is updated every ten minutes. The recorded maintenance time data is shown in Figure 2, where the rows represent the time of the 10-min cycles and the columns represent the wind turbine number. If the recorded time data equal “NAN”, it means that the wind turbine is under maintenance. To better describe the maintenance time data, attributes such as NAN time, time condition, and NAN period are defined. Figure 3 illustrates that the maintenance data contains three attributes: NAN time, time condition, and NAN period. “NAN time” is the maintenance time of the wind turbine, shown as a green double-headed arrow. “Time condition” is the interval from the end of the previous NAN to the start of the current NAN, shown as a blue double-headed arrow. “NAN period” is the interval from the end of the previous NAN to the end of the current NAN, shown as an orange double-headed arrow. Figure 4 shows an example of these attributes being extracted through data aggregation. Starting from the left are the columns of wind turbine number, the previous NAN end time, the current NAN start time, the current NAN end time, and the interval of the NAN period. In Table 2, sensors would record the fault message represented as “event code” when the abnormal operation of a wind turbine occurs. The Vestas supervisory control and data acquisition (SCADA) system would collect and record the event code that occurs first in that 10-min cycle time.

**Figure 5:**
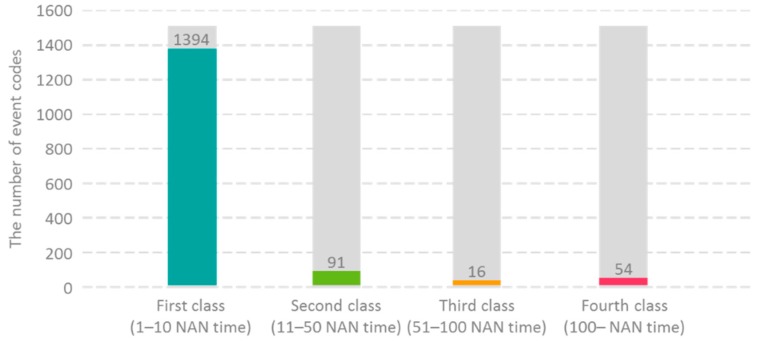
The number of event codes with regard to maintenance categories. (*x*-axis indicates four categories of NAN periods and their corresponding length of NAN times; *y*-axis is the number of event codes).

The NAN period is classified into four maintenance categories according to the length of its NAN time, and the fourth class is defined as the long cycle maintenance time because it has the longest maintenance hours. The NAN period classification rule is defined as follows. The length of the NAN time in the NAN period ranging from 1–10 is classified as the first class, 11–50 as the second class, 51–100 as the third class, and those above 100 as the fourth class. Figure 5 shows the relationship between the maintenance categories and the number of event codes. Apparently, the first class is the most frequently observed one, so it exhibits the highest number of event codes.

**Figure 6:**
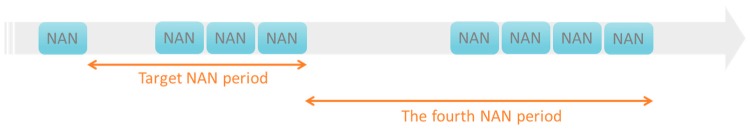
Time graph of the relationship between the target NAN period occurring before the NAN period of the fourth class.

The fourth class has the longest maintenance hours among the four maintenance categories, which means that its maintenance cost is the highest. If the long cycle maintenance time can be predicted, the cost could be significantly reduced. We analyze the event codes of the NAN period that occurred before the fourth class. Figure 6 is an example of the time graph of the target NAN period and the NAN period of the fourth class.

It is quite reasonable to assume that several event codes exist, which lead to long cycle maintenance. We use the Apriori algorithm to find association rules of the event codes of the NAN period occurring before the fourth class. Because the operation performance of two wind farms is different, we analyze the maintenance data of two wind farms separately. From the analysis results, we found that two event codes, Code 154 and Code 309, happened along with the long cycle maintenance time. Based on knowledge in this domain and the experience of the Taiwan Power Company, another two event codes, Code 147 and Code 214, are also considered to be related to the long cycle maintenance time. So, the frequency of Code 154, Code 309, Code 147, and Code 214 are chosen to be the features for predicting the long cycle maintenance time.

**Figure 7:**
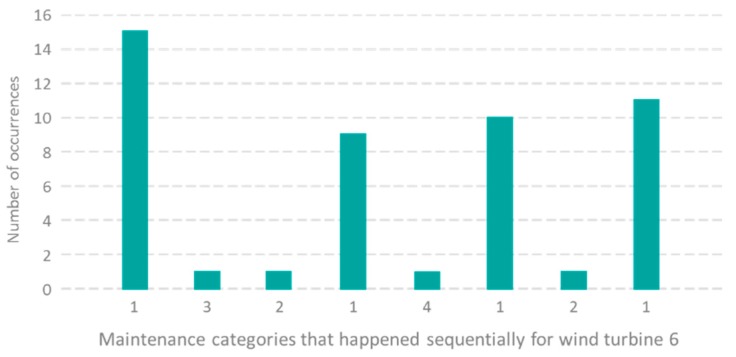
Histogram of the number of occurrences with regard to maintenance categories. (*x*- and *y*-axes are maintenance categories that happened sequentially, and the number of occurrences, respectively).

To find other features, we designed a rule to predict the long cycle maintenance time. If there are more than eight consecutive short NAN periods before the fourth class, and the time between the fourth class and the previous NAN period is shorter than the predefined threshold, a large maintenance cycle is likely to occur. Figure 7 shows that for the wind turbine 6, a total of twenty-six consecutive short NAN periods (15—first class, one—third class, one—second class, and nine–one class) occur before the fourth class. In Table 3, time thresholds are set to be less than 14 days, 30 days, or 45 days, and the prediction accuracy can reach 30%. The result is satisfying when we use the rule to predict the long cycle maintenance time only. It means that there is a high correlation between the number of consecutive short NAN periods and the long cycle maintenance time. Therefore, the number of consecutive short NAN periods is chosen to be the feature to predict long cycle maintenance time.

#### 3.1.2. Operation Data

Operation data including wind speed, the power output of wind turbines, the oil temperature of the wind turbine gearbox, and the temperature of the high-speed bearings, are collected by the Vestas supervisory control and data acquisition (SCADA) system. The operation data is collected for 941 consecutive days, and the updating frequency is every ten minutes, which is the same time length as the available maintenance data. There are some errors in the wind speed and the power output of the wind turbine’s data due to the bug in the data collection system. The relation of wind speed and output power could be easily modeled by regression analysis. According to the characteristics of linear regression, linear regression is a suitable model to fit this formula. To filter the errors in the wind speed and the power output of the wind turbines data, a residual threshold is set. Then, the data whose residual is greater than the threshold is filtered. Most of the error data could be filtered after this step. We calculate the standard deviation from the predicted moment to the last long cycle maintenance. The standard deviation is chosen to be the feature of the long cycle maintenance time.

### 3.2. Prediction Model Construction

To predict the long cycle maintenance time precisely, we proposed a hybrid network which combines the CNN and SVM. CNNs are quite good at learning invariant features, but not always optimal for classification (most of the trainable parameters are in the middle layers). On the other hand, SVM with a fixed kernel function, cannot learn complicated invariances but can produce good decision surfaces when applied to well-behaved feature vectors. It is interesting to investigate a hybrid system in which the convolutional net is trained to extract features that are relatively invariant to irrelevant variations of the input. Thus, an SVM with a simple Gaussian kernel can perform well at separating the categories in the learned feature space. SVM is added to the top layer, instead of the softmax layer.

**Figure 8:**
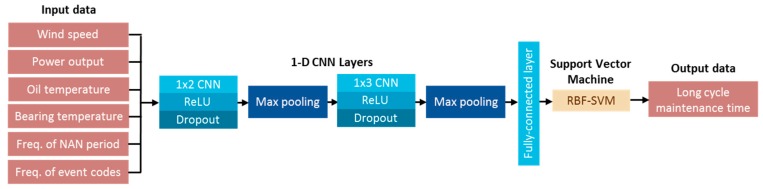
The architecture of the hybrid network.

The architecture of the hybrid network is shown in Figure 8, where two-layer CNNs are used to extract features, and the features are then used as the input to train an RBF-SVM (Radial Basis Function). Six features: wind speed, power output of the wind turbines, oil temperature for the wind turbine gearbox, temperature of the high-speed bearings, the number of consecutive short NAN periods, and the frequency of event codes (Code 154, Code 309, Code 147, and Code 214) from the data aggregation are used to train the hybrid network. In the following experiments, a two-layered 1-D CNN and the fully connected layer are used to extract features, and the features are then used as input to train the RBF-SVM. The model is trained by the features extracted from the data aggregation.

## 4. Experimental Results

Data aggregation and model construction are implemented in Python with Keras [20]. We used two 1-D CNNs in all experiments with SVM in the output layer. The experiments conducted in the proposed method used the dataset collected from sensors in 31 wind turbines for 941 consecutive days. This dataset is a collection of wind turbine operation data, maintenance time data, and event code data. There are 4,205,119 records in each of the operation data of the four categories: wind speed, the power output of the wind turbines, the oil temperature for the wind turbine gearbox, and the temperature of the high-speed bearings. The maintenance time data also contains 4,205,119 samples and the event code data contain 6251 samples. We used data aggregation to extract the input data of size 6 × 135,649 from six features, including the above four categories, the number of consecutive short NAN periods, and the frequency of event codes. The dataset is randomly split into two parts, 80% as training data and 20% as test data. Both the first 1 × 2 CNN and the second 1 × 3 CNN consisted of a rectified linear unit (ReLU) and a dropout rate of 0.3. The maximum pooling layer with a stride of two is placed after each CNN, so that the features are downscaled twice to reduce the computational cost. The size of the fully-connected layer is 1 × 128. The output layer used RBF-SVM for classification. The learning rate ε is set at 0.001. To prevent the underestimation or overestimation, the validation loss is ensured to be similar and slightly higher than the training loss. This step can assure that the prediction model is robust not only for the training data. Based on the above settings, a well-trained hybrid network can be obtained.

Classification accuracy is chosen as the machine learning metric to evaluate our supervised learning model. Table 4 is the class corresponding to accuracy. In this experiment, we assume that the maximum long cycle maintenance time interval is 900 days. The classes are the number of segments that 900 days are divided into. For example, we set the prediction system output to binary classification. The 900 days will be divided into two segments (Class 2): the first segment is 0–450 days and the second is 451–900 days. If we get an output which is the first one, it means that the long cycle maintenance time is predicted within 0–450 days. If the output is the second, the long cycle maintenance time is predicted within 451–900 days. For Class 3, the 900 days are divided into three segments, 0–300, 301–600, and 601–900 days; the same method applies to Class 4–Class 9. It is clear that the more the classes, the lower the accuracy. The accuracy of Class 9 reaches 73%, e.g., the prediction accuracy of our method is higher than 70% for the long cycle maintenance within 100 days, which can enhance the wind turbine management for power companies.

## 5. Conclusions

This paper proposes a machine learning framework to predict the long cycle maintenance time of wind turbines efficiently. In addition to the sensor data, we also analyze the characteristics of event codes to extract two useful features including the frequency of the event code and the NAN period, which enhances the prediction performance. Different from the traditional CNN, the hybrid network, which is based on two layers of 1-D CNN and the RBF-SVM, benefits from invariant features learned from CNN and an accurate classification output predicted by SVM. The hybrid network achieves better prediction results for the long cycle maintenance time of the wind turbine. Experimental results show that the prediction accuracy of our method is higher than 70% for the long cycle maintenance within 100 days, which can really enhance the wind turbine management for power companies. In the future, we will make use of more data from sensors to discover insightful information for event prediction and further enhance the maintenance of wind turbines.

## Figures and Tables

**Table 1:** Specification of the V80 module wind turbine.

**Operating Data**	Rated power	2000 kW
	Cut-in wind speed	4.0 m/s
	Rated wind speed	15 m/s
	Cut-out wind speed	25 m/s
**Rotor**	Rotor diameter	80 m
	Operational interval	9–19 rpm
	Air brake	Full blade feathering with 3 pitch cylinders
**Gearbox**	Two planetary stages and one helical stage	

**Table 2:** Event codes of failure messages in common use.

Event Codes	Failure Message
900	Pause pressed on the keyboard
276	Start auto-outyawing CCW
329	DC Overvolt: 880 V state 1
144	High wind speed: 25.1 m/s
315	ExEx low voltage L1: 282 V
340	OVP active UDC 0V state 1
335	ExtHighIRotorInv phase: 3
147	High gear temp
154	Max rotor RPM: 21.8 RPM
214	Low oil level
309	Pause over RCS 13

**Table 3:** The accuracy of the long cycle maintenance time prediction with the rule.

Time Condition	Less than 14 Days	Less than 30 Days	Less than 45 Days
Rule	29.79%	31.91%	31.91%

**Table 4:** The prediction accuracy of long cycle maintenance time.

Class	Accuracy
2	95%
3	92%
4	87%
5	84%
6	76%
7	77%
8	76%
9	73%
